# Quantitative Measurement of Cooperative Binding in Partially Dissociated Water Dimers at the Hematite “R-Cut” Surface

**DOI:** 10.1021/acs.jpcc.4c04537

**Published:** 2024-09-30

**Authors:** Paul T. P. Ryan, Panukorn Sombut, Ali Rafsanjani-Abbasi, Chunlei Wang, Fulden Eratam, Francesco Goto, Cesare Franchini, Ulrike Diebold, Matthias Meier, David A. Duncan, Gareth S. Parkinson

**Affiliations:** † Institute of Applied Physics, Technische Universität Wien, 1040 Vienna, Austria; ‡ Diamond Light Source, Harwell Science and Innovation Campus, OX11 0QX Didcot, U.K.; § Politecnico di Milano, Piazza Leonardo da Vinci, 20133 Milano MI, Italy; ∥ Faculty of Physics and Center for Computational Materials Science, University of Vienna, 1040 Vienna, Austria

## Abstract

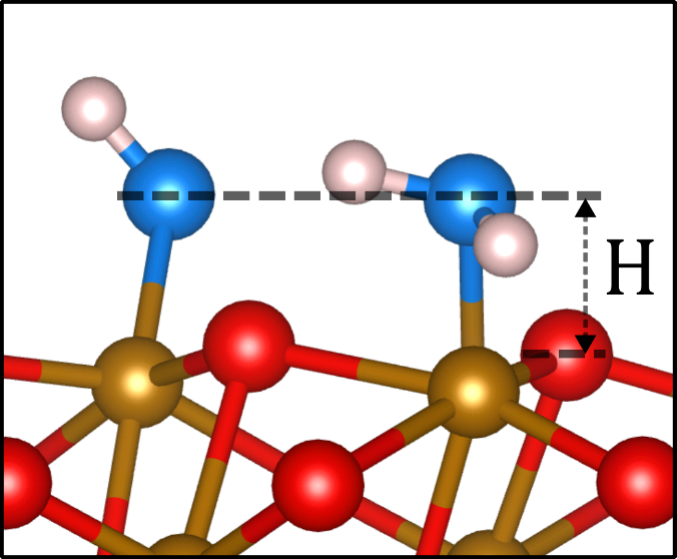

Water–solid interfaces pervade the natural environment and modern technology. On some surfaces, water–water interactions induce the formation of partially dissociated interfacial layers; understanding why is important to model processes in catalysis or mineralogy. The complexity of the partially dissociated structures often makes it difficult to probe them quantitatively. Here, we utilize normal incidence X-ray standing waves (NIXSW) to study the structure of partially dissociated water dimers (H_2_O–OH) at the α-Fe_2_O_3_(012) surface (also called the (11̅02) or “R-cut” surface): a system simple enough to be tractable yet complex enough to capture the essential physics. We find the H_2_O and terminal OH groups to be the same height above the surface within experimental error (1.45 ± 0.04 and 1.47 ± 0.02 Å, respectively), in line with DFT-based calculations that predict comparable Fe–O bond lengths for both water and OH species. This result is understood in the context of cooperative binding, where the formation of the H-bond between adsorbed H_2_O and OH induces the H_2_O to bind more strongly and the OH to bind more weakly compared to when these species are isolated on the surface. The surface OH formed by the liberated proton is found to be in plane with a bulk truncated (012) surface (−0.01 ± 0.02 Å). DFT calculations based on various functionals correctly model the cooperative effect but overestimate the water–surface interaction.

## Introduction

Metal oxide surfaces are omnipresent in the environment, and their interaction with water underlies natural processes such as geochemistry, corrosion, and cloud formation. Metal oxides are also often employed as catalysts, catalytic supports, and electrocatalysts, and it is known that adsorbed water affects the catalytic process even in cases where it is not directly a reactant.^1^ For example, water adsorbed at the oxide surface affects the morphology and reactivity of supported metal adatoms or clusters,^2−5^ and metal oxides utilized as electrocatalysts can undergo hydroxylation and oxygen exchange reactions with the water.^6−9^ Correctly modeling the water–oxide interaction is therefore an important issue and a prerequisite for understanding how these materials behave under realistic application conditions.

**Figure 1:**
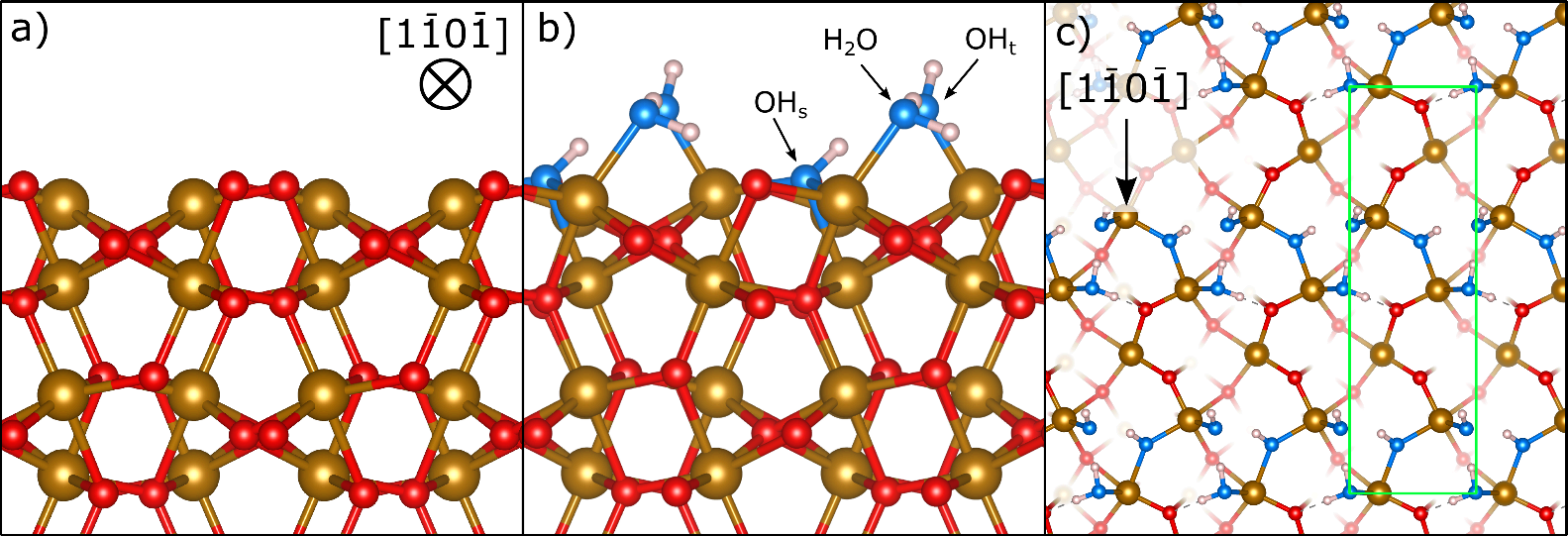
Model for water adsorption on the α-Fe_2_O_3_(012)-(1 × 1) surface.^20^ a) Side view of the clean bulk-truncated α-Fe_2_O_3_(012)-(1 × 1) surface. b) Side view of the DFT model showing the H_2_O–OH_t_ dimer and OH_s_. The surface atoms have negligible vertical relaxation. c) Top view of the DFT model with a green (1 × 3) unit cell. Red and brown atoms are O anions and Fe cations of the bulk, respectively. Blue atoms are the oxygens of the H_2_O, OH_t_, and OH_s_ species, and white atoms are hydrogens.

Hematite (α-Fe_2_O_3_) is a naturally abundant mineral that has shown promising potential in the context of photochemical water splitting. It has a 2 eV band gap, which facilitates oxygen evolution using visible light.^10−12^ Recently, hematite has found use as a support for so-called single-atom catalysts for reactions including the water–gas shift reaction and the (electrochemical) oxygen reduction reaction.^13−16^ The α-Fe_2_O_3_(012)-(1 × 1) surface (also called (11̅02) or “R-cut” surface) is one of the most prevalent low-index facets, and water adsorption has been studied previously in both ultrahigh vacuum (UHV)^17−19^ and in liquid.^6,20,21^ All studies to date suggest that water exposure leads to both molecular and dissociated components at the interface. Recently, we studied water adsorption on this surface^20^ using noncontact atomic force microscopy (nc-AFM), X-ray photoelectron spectroscopy (XPS), and density functional theory (DFT)-based calculations and concluded that the surface stabilizes H_2_O–OH dimers. More specifically, it was found that isolated H_2_O molecules adsorb intact at a surface cation, but the interaction with a second H_2_O leads to its dissociation into a terminal hydroxyl (OH_t_) adsorbing at a neighboring cation site and an additional surface hydroxyl (OH_s_) species at a lattice oxygen on the surface. The H_2_O and OH_t_ form a hydrogen bond leading to a partially dissociated dimer (H_2_O–OH_t_) as shown schematically in Figure 1 b and c.

The phenomenon of partial dissociation has been reported previously for several metal oxide^22−28^ and metal^29−31^ surfaces. It occurs when the energy gained through the formation of a H_2_O–OH hydrogen bond compensates for the energy lost creating the less favorable adsorbate (in isolation). The interaction can be further strengthened by a so-called “cooperative binding” effect^32−34^ in which it is assumed that water molecules optimally donate and receive equally in their bonding interactions. Thus, a stronger intermolecular hydrogen bond is accompanied by a stronger surface bond between the water and the surface, which should manifest as shorter water–cation bond lengths. This is the effect that we aimed to directly measure in this article.

The structure of the substrate is important for observing partial dissociation and cooperative binding because undercoordinated cation–anion pairs are required to host the H_2_O and OH groups. Also, the cation–cation distance must be short enough to facilitate the formation of a strong hydrogen bond between molecular H_2_O and the terminal OH_t_. Complicated arrangements can occur on surfaces where the H_2_O and OH groups form overlayers with large unit cells,^22,23^ which makes the elucidation of the structure challenging. DFT-based calculations are often utilized, but modeling such systems is difficult due to the subtle balance of the interactions involved and the need to account for dispersion interactions. In contrast, the H_2_O/α-Fe_2_O_3_(012)-(1 × 1) system is unusual in that it limits the size of partially dissociated agglomerates to H_2_O-HO_t_ pairs, offering a comparatively simple system for interrogation of the cooperative binding effect.

In this study, we utilize the quantitative structural technique normal incidence X-ray standing waves (NIXSW) to chemically resolve the adsorption sites of species in the H_2_O–OH dimer on α-Fe_2_O_3_(012)-(1 × 1). Our results show that the H_2_O and OH_t_ groups reside at the same height above the surface (1.45 ± 0.04 and 1.47 ± 0.02 Å, respectively), which implies a similar Fe–O bond length for the H_2_O and OH_t_ (∼2.0 Å). These results agree with the results of our prior DFT calculations and corroborate the picture of partially dissociated water dimers originally derived from noncontact atomic force microscopy (nc-AFM) images. Also, they provide compelling evidence for the involvement of cooperative binding effects for water adsorption on metal oxide surfaces. Such observations are found in prior studies,^22−27^ though to our knowledge our results represent the first quantitative measurement of this cooperative binding effect.

## Experiment and Methods

### Samples

A polished α-Fe_2_O_3_(012) “R-cut” surface single crystal (±0.1°, from the SurfaceNet GmbH) was prepared *in situ* via several cycles of sputtering (Ar^+^, voltage 1 keV, emission current 3 μA, 30 min) and annealing in 2 × 10^–6^ mbar of oxygen (∼500 °C, 30 min). The prepared samples showed a (1 × 1) LEED pattern consistent with a bulk-truncated surface.^18,19^ A side view of this surface structure is shown in Figure 1 a.

High-purity deionized water was obtained from a Milli-Q system and cleaned *in situ* by several freeze–pump–thaw cycles. The clean α-Fe_2_O_3_(012)-(1 × 1) surface was exposed to 3 × 10^–8^ mbar of water for 10 min at 300 K (∼14 L, where 1 L is 1 × 10^–6^ mbar·s). Following transfer into the analysis chamber (within 5–10 min), the sample was cooled to 200 K using a liquid nitrogen cryostat. Based on our previous study, this preparation procedure should produce a (1 × 3) overlayer of partially dissociated water dimers, as shown in Figure 1 b and c.^20^

### NIXSW and SXPS

The NIXSW technique exploits the X-ray standing wave formed by the interference between the incident and reflected waves around the Bragg condition for a given reflection (*h*, *k*, *l*).^35−37^ The standing wave’s period matches the interplanar spacing *d*_*hkl*_ between the Bragg diffraction planes.^38^ The standing wave’s phase, and thus the location of its maximum intensity, varies as the photon energy is scanned through the Bragg condition. When the phase is π, the maximum intensity is halfway between the Bragg diffraction planes; when the phase is zero, the maximum intensity coincides with the Bragg diffraction planes. Any atom within this standing wavefield will therefore experience a varying electromagnetic field intensity as a function of its position between these Bragg diffraction planes. This variation in intensity results in a characteristic absorption profile, which can be acquired by monitoring the relative photoelectron yield. The measured profile is then fitted uniquely, using dynamical diffraction theory,^39^ by two dimensionless parameters: the coherent fraction, *f*_*hkl*_, and the coherent position, *p*_*hkl*_. These correspond to the degree of order and the mean position of the absorber atoms relative to the Bragg diffraction planes, respectively.^36,37^ When the origin of the substrate atomic coordinates is chosen to be in the surface plane, the coherent position is related to the mean adsorption height (*H*) by
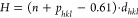
1where *d*_*hkl*_ is the reflection layer spacing and *n* is an integer which relates to so-called “modulo-d” ambiguity,^36^ where adsorption heights that differ by the interplanar spacing cannot be directly differentiated. In practice, however, the correct value of *n* can often be easily assigned as *d*_*hkl*_ typically is on the order of ∼2 Å; thus, it is generally trivial to exclude adsorption heights that are unphysically low or high. Since we utilize only the (024) reflection here, *d*_024_ = 1.84 Å, and the coherent fraction and coherent position are denoted as *f*_024_ and *p*_024_, respectively. Note that because of the standing wave being generated by the crystallinity of the bulk substrate, the adsorption height measured in NIXSW is not relative to the position of the outermost atoms at the surface but rather to a projected bulk-like termination of the surface. To obtain adsorption heights relative to the bulk-like surface O atoms, the coherent position of the surface O atoms (0.61) has been subtracted from *p*_*hkl*_ in eq 1. Our DFT calculations indicate that the terminal O atoms have a negligible vertical surface relaxation after the adsorption of water (see Table 1), thus making *H* a direct measure of the adsorption height with respect to the surface oxidic O atoms.

**Table 1:** DFT Heights (*H*_H_2_O_, *H*_OH_t__, and *H*_OH_s__) of the Species in the H_2_O–OH_t_ Dimer with a Comparison to the NIXSW Results of Model 2^a^

	*H*_H_2_O_ (Å)	*H*_OH_t__ (Å)	*H*_OH_s__ (Å)	Δ*H*_Os_ (Å)	Δ*H*_FeH_2_O_ (Å)	Δ*H*_FeOH_ (Å)	*E*_ads_ (eV)	Δ*c* (Å)
Model 2	1.45(4)	1.47(2)	–0.01(2)	-	-	-	-	-
Models 1–4	-	1.38–1.47	–0.01–0.09	-	-	-	-	-
								
HSE 12%	1.42	1.50	0.11	0.00	+0.06	+0.10	–1.19	0.06
HSE 25%	1.36	1.44	0.06	–0.04	+0.02	+0.10	–1.30	–0.01
OptB88-DF	1.42	1.47	0.11	–0.02	+0.04	+0.10	–1.60	0.02

a For the *H*_OH_t__ and *H*_OH_s__ results, the range of values for models 1–4 (see Section 2 of the SI) are also given. These DFT heights are calculated with respect to an oxygen bulk-terminated (012) surface. Values in parentheses are the error in the last significant figure. Δ*H*_Os_ is average change in vertical height of the surface oxygens after water exposure. Δ*H*_FeH_2_O_ and Δ*H*_FeOH_ are the vertical height changes of the Fe atoms bound to the H_2_O and OH_t_ species (compared to the dry bulk surface). *E*_ads_ is the calculated adsorption energy of the H_2_O. Δ*c* is the difference between the experimental and calculated *c* unit cell parameters for α-Fe_2_O_3_.

By acquiring the photoelectron yield from the O 1s core level as a probe of the NIXSW absorption rate, we obtain a chemically resolved probe that permits signals from the bulk oxide, surface hydroxide, and adsorbed water to be discriminated independently.

All measurements were conducted in the permanent ultrahigh vacuum (UHV – ∼1 × 10^–10^ mbar) end station on the I09 beamline^40^ at the Diamond Light Source. Beamline I09 utilizes two separate undulators, which are monochromated separately by a double Si(111) crystal monochromator and a plane grating monochromator. These two separate lines provide simultaneous access to both “hard” and “soft” X-ray energies, respectively. Specifically, we have used incident photon energies of 650 eV for all of the O 1s soft X-ray photoelectron spectroscopy (SXPS) measurements. For the NIXSW (024) reflection, a hard photon energy range of 3350–3370 eV was used. The absolute binding energy scales of all XP spectra were calibrated by subsequent measurements of the Au 4f core level from a gold foil situated below the sample holder.

All photoelectron spectra were acquired using a VG Scienta EW4000 HAXPES hemispherical electron analyzer (angular acceptance range ±28°) mounted perpendicular to the incident radiation and in plane with the polarization of the incident photon (linear-horizontal). All photoelectron spectra were peak fitted by using a numerical convolution of a Lorentzian and a Gaussian peak profile. For all peaks in all spectra, the same Lorentzian peak width was used, as determined from the fits of the bulk oxide O 1s photoemission peak, and the Gaussian peak width was allowed to vary. A Shirley background^41^ was subtracted from each spectrum.

### Theoretical Details

We utilized the Vienna *ab initio* Simulation Package (VASP)^42,43^ with the optB88-DF^44,45^ functional utilizing a *U*_eff_ of 5 eV.^46^ Additionally, we investigated a hybrid functional (HSE06) with the fractions of exact exchange of 12% and 25% and a range separation parameter of 0.2^–1^ Å^–1^. A further set of functionals were tested (in total 20), and details and citations for these other functionals are found in Section 1 of the SI.

The surface calculations employed symmetric slabs with only the two inner central O layers kept fixed. The model of a (1 × 3) overlayer of partially dissociated water (H_2_O–OH) dimers contains four water molecules per (1 × 3) unit cell (Figure 1 b and c).^20^ Two H_2_O molecules are molecularly adsorbed on Fe cation sites, and two H_2_O molecules dissociate, liberating two protons to two surface oxygen atoms to form two OH_s_ species in the O surface plane and two OH_t_ species terminal to surface Fe cations. This model is derived from our prior nc-AFM experimental study.^20^ Between neighboring H_2_O–OH_t_ dimers along the (01̅01̅) direction, one Fe cation site is left vacant. Note that the adsorption site atop a surface Fe cation is where the next O atoms would reside if the bulk corundum structure were continued outward, although in that case the next layer of the bulk structure would have a larger height (1.62 Å) than that found for OH_t_/H_2_O (∼1.46 Å). Further details of the computational setup are provided in Section 1 of the SI.

The average adsorption energy per H_2_O molecule (*E*_ads_) is computed according to the formula
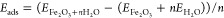
2where *E*_Fe_2_O_3_+*n*H_2_O_ is the total energy of the α-Fe_2_O_3_(012) surface with adsorbed H_2_O, *E*_Fe_2_O_3__ is the total energy of the clean α-Fe_2_O_3_(012) surface, *E*_H_2_O_ represents the energy of the H_2_O molecule in the gas phase, and *n* is the number of H_2_O molecules.

The O 1s core-level binding energies are calculated in the final state approximation.^47^ The calculation was undertaken with respect to oxygen in the bulk position.

To elucidate the underlying cooperative binding mechanism, we calculated the total variation of charge transfer (Δ_Tot_) between the on-the-surface adsorbed H_2_O–OH_t_ dimer and the on-the-surface adsorbed individual molecules. Δ_Tot_ is defined as
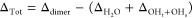
3with
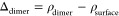
4
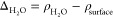
5
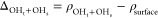
6where ρ_*x*_ is the electronic charge distribution given by DFT for configuration *x*. Δ_Tot_ can thus be recast as
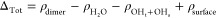
7where the surface is needed once to compensate for the double counting of it in the individual-molecule interactions. A positive value can therefore be attributed to stronger bonding between the molecules involved in the dimer. The individual-molecule components are calculated at partially dissociated water dimer positions (no relaxations allowed) but with the counter-component in the dimer removed.

## Results

### SXPS

**Figure 2:**
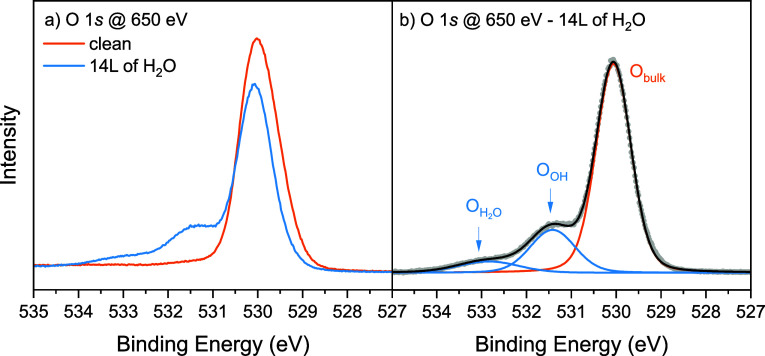
SXPS results. a) O 1s SXPS core-level spectrum of the clean α-Fe_2_O_3_(012)-(1 × 1) surface and after exposing this surface to 14L of H_2_O. b) The same spectrum from a) after dosing H_2_O but peak fitted with three components assigned as oxygen from the bulk crystal, O_bulk_, adsorbed hydroxyls, O_OH_, and adsorbed water, O_H_2_O_.

Figure 2a shows the O 1s SXPS spectra for the clean α-Fe_2_O_3_(012)-(1 × 1) surface and for the surface after exposure to 14 L of water vapor at 300 K (measured at 200 K). Upon exposure to water, two new photoemission peaks are visible at higher binding energies than the main bulk oxide peak. The bulk O 1s peak is also found to shift to higher binding energies. This has been observed previously and is attributed to band bending.^20^Figure 2b shows the peak-fitted spectrum from Figure 2a after exposure to water. Three peaks were used in the fitting and assigned as oxygen from the bulk oxide, O_bulk_, oxygen from adsorbed hydroxides, O_OH_, and oxygen from adsorbed water, O_H_2_O_.

The binding energies of the O_H_2_O_ and O_OH_ peaks correspond well to prior XPS studies of adsorbed water and hydroxides on metal oxides.^22,48−51^ Our DFT calculations also show that the OH_t_ and OH_s_ species have an O 1s core-level binding energy that differs by only ∼0.1 eV, which is within the error of the calculation. Comparison of the O_H_2_O_ and O_OH_ relative peak areas shows that there is 21% more OH than expected. From the model of our prior nc-AFM study,^20^ one would expect exactly double O_OH_ vs O_H_2_O_. The 21% increase in the OH population is most likely from a number of different sources. A nonexhaustive list could include extra dissociative adsorption at defects or step edges, dissociative adsorption of singularly adsorbed H_2_O, and extra OH intensity due to slight carbon contamination (see Section 2 of the SI). We exclude beam damage as a source; no beam damage was observed during the measurements, and the extra OH intensity is comparable to out prior laboratory-based XPS study (∼25%).^20^

### NIXSW

**Figure 3:**
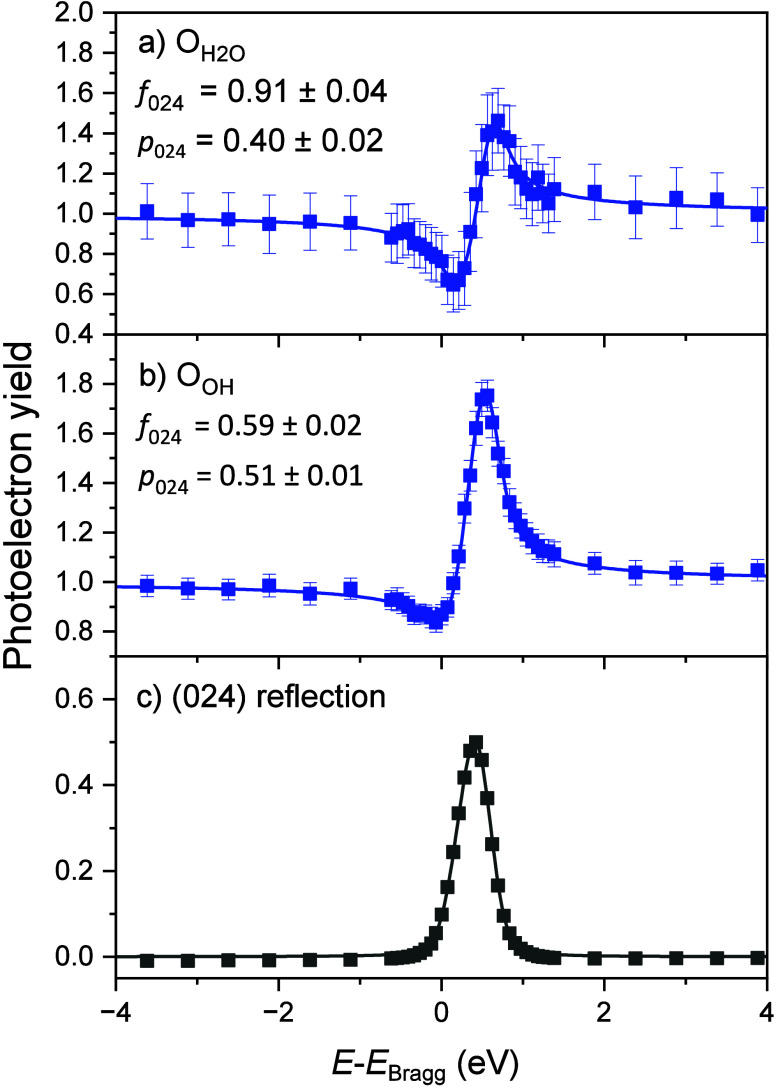
NIXSW results. Photoelectron yield profiles for the a) O_H_2_O_ and b) O_OH_ photoemission peaks and c) the intensity of the (024) reflection. Given are the fitted coherent fraction, *f*_024_, and the coherent position, *p*_024_, for each absorption profile.

Figure 3 shows the NIXSW photoelectron yield profiles for the O_OH_ and O_H_2_O_ oxygen as well as the measured intensity of the (024) reflection. The fitted coherent fraction of the O_H_2_O_ photoemission peak (*f*_024_ = 0.91 ± 0.04) implies that the H_2_O occupies an extremely well-defined position normal to the (012) surface, with this position defined by the fitted coherent position (*p*_024_ = 0.40 ± 0.02). The small deviation from unity in the coherent fraction can be attributed solely to molecular and crystal vibrations.^52^

**Figure 4:**
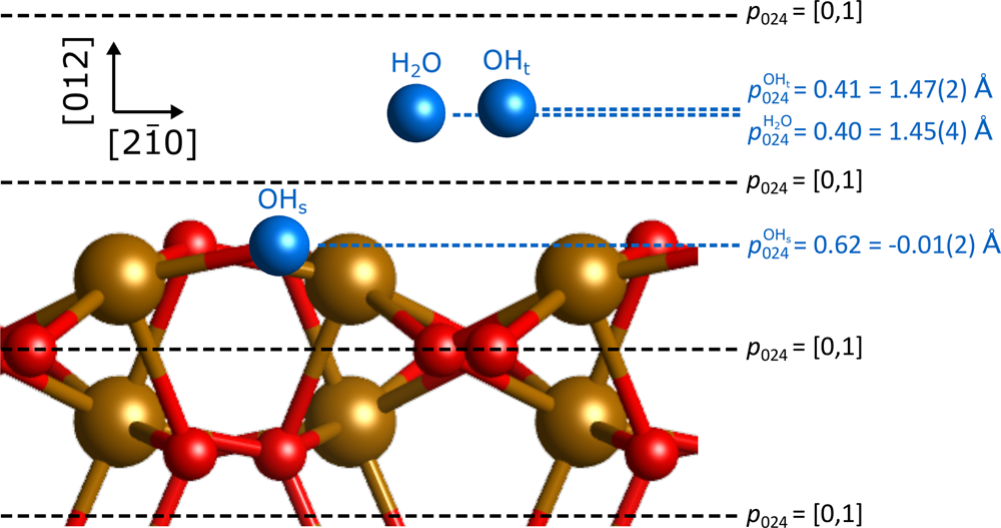
Schematic showing the positions of H_2_O, OH_t_, and OH_s_ in blue projected onto a bulk truncated (012) surface. The (024) reflection periodicity is shown by the black dashed lines. Given in blue are the measured or calculated coherent positions, *p*_024_, for each species and the corresponding heights above a (012) bulk oxygen surface layer. In parentheses is the error. Note that this schematic does not take into account the lateral placement of the H_2_O and OH_t_, and this has been chosen as oxygen bulk (continuation) sites.

As it is assumed that the H_2_O adsorbs above the surface, only values of *n* > 1 in eq 1 are considered for the H_2_O. Should *n* ≥ 2 be considered, the adsorption height of the H_2_O would be unphysically large (>3 Å). Thus, it is assumed that *n* = 1 and the adsorption height of H_2_O above the bulk-like terminated α-Fe_2_O_3_(012) surface is *H*_H_2_O_ = 1.45 ± 0.04 Å, close to a bulk continuation adsorption height of 1.61 Å. This is schematically shown in Figure 4.

The fitted coherent fraction of the O_OH_ photoemission peak (*f*_024_ = 0.59 ± 0.02) is significantly lower than that of the O_H_2_O_. Such a low coherent fraction (<0.8) cannot be attributed to molecular or crystal vibrations alone,^52^ so the O_OH_ photoemission peak must correspond to chemically similar oxygen atoms located at different distinct heights in the [024] direction. This makes sense because water dissociation is expected to produce both an OH_t_ adsorbed above a surface cation and an OH_s_ at a surface oxygen atom.^20^

It is possible to extract the two individual OH adsorption heights by making reasonable assumptions about OH_s_ and OH_t_. We assume that the 21% excess O_OH_ signal contributes decoherently to the OH position, giving an order parameter^53^ of *C* = 0.79 (see SI Section 2 for further explanation). This would be the case if the extra OH intensity came from a number of sources, such as dissociative adsorption at defects and step edges or slight carbon contamination. Also, we assume a similar reduction of *f*_024_ from thermal vibrations as found for the O_H_2_O_ species (Debye–Waller factor = 0.91). Both of these factors lead to a true structural *f*_024_ = 0.82.^53^ Finally, by assuming an equal occupation of OH_t_ and OH_s_, the analysis of the fitted coherent position (*p*_024_ = 0.51 ± 0.01) leads to an OH_t_ coherent position of *p*_024_ = 0.41 ± 0.01 and an OH_s_ coherent position of *p*_024_ = 0.62 ± 0.01.

A more detailed description of the calculation is provided in Section 2 of the SI. The results from other models, obtained by varying the relative population of the OH_t_ and OH_s_ sites, the order parameter *C*, and the Debye–Waller factor, are also provided in Section 2 of the SI (models 1–4). Generally, the physically reasonable models in the SI have results close to the model presented here (model 2). While there may be some ambiguity on the precise adsorption height of both OH_s_ and OH_t_, all of the models not excluded by our prior nc-AFM measurements indicate that the OH_s_ species is effectively in plane with the surface oxygen atoms and that the OH_t_ species is effectively in plane with the water molecule at the approximate height of the O bulk continuation site.

Using eq 1, we can calculate heights for the two OH species with respect to a (012) bulk truncated oxygen surface layer for model 2. Following the same arguments as for H_2_O, *n* = 1 for OH_t_ and *n* = 0 for the OH_s_. In turn, we find that the OH_t_ sits at a height *H*_OH_t__ = 1.47 ± 0.02 Å above the surface, in plane with the adsorbed H_2_O. The OH_s_ is found in plane with the surface oxygens (*H*_OH_s__ = −0.01 ± 0.02 Å). These heights are schematically shown in Figure 4. Note that the lateral placement of the species is not determined here and for the schematic has been defined as oxygen bulk (continuation) sites.

### DFT

Table 1 compares the results of the DFT calculations for the H_2_O–OH_t_ dimer with the measured experimental adsorption heights (*H*_H_2_O_, *H*_OH_t__, and *H*_OH_s__) from the NIXSW. These heights were calculated in the same manner in which the NIXSW measurements are undertaken by projecting the species’ position onto a bulk unit cell. This ensures that any bulk or surface relaxations are taken into account, and because the NIXSW measurement is not sensitive to any bulk or surface relaxations,^37^ this allows for direct comparison with the NIXSW results.

The optB88-DF, HSE 12% and 25% functionals were selected for comparison to the NIXSW results because they reproduce the bulk lattice parameters of the α-Fe_2_O_3_ crystal extremely well. This is demonstrated by Δ*c* in Table 1, which is the difference between the experimental and optimized *c* lattice parameters for each functional. Note that in the actual calculations presented here the experimental lattice parameters were used so that comparison to the NIXSW measurements could be made. We have also calculated the same results for other functionals, and these are given in Section 3 of the SI along with further detailed discussions of all of the theoretical results. By undertaking correlational analysis with all of the tested functionals, we find that all of the structural values (i.e., both heights and bond lengths) correlate extremely strongly with the Δ*c* parameter (correlation coefficients >0.9, Table S3). This reinforces our choice of optB88-DF, with HSE 12% and 25% calculations being the most suited for comparison with the NIXSW results.

In terms of *H*_H_2_O_, all three functionals overbind the position of H_2_O (Table 1). However, we believe the OptB88-DF functional performs best overall, placing the H_2_O and OH_t_ almost coplanar, as determined by the NIXSW measurements. The hybrid functionals perform poorly in this regard, placing the OH_t_ consistently too high with respect to the H_2_O species. In all cases, both the H_2_O and OH_t_ adsorption heights and Fe–O bond lengths are of similar order, between 1.4 and 1.5 Å (for heights) and 1.9 and 2.1 Å (for bond lengths; Table 2). Our DFT calculations also show that vertical relaxations of the surface Fe atoms bound to the H_2_O and OH_t_ species, Δ*H*_FeH_2_O_ and Δ*H*_FeOH_ (Table 1), are at most ∼+ 0.05 and ∼+ 0.1 Å, respectively. These relaxations would imply very similar bond lengths for the Fe–H_2_O and Fe–OH_t_ bonds, and this is indeed the case (Table 2); the +0.1 Å upward relaxation of the Fe bound to OH_t_ compensates for the higher position of the OH_t_.

**Table 2:** DFT Fe–O Bond Lengths for the Species in the H_2_O–OH_t_ Dimer (*d*_Fe–H_2_O_ and *d*_Fe–OH_t__) and for Isolated Species (*d*_Fe–H_2_O_^iso^ and *d*_Fe–OH_t__^iso^) on the α-Fe_2_O_3_(012)-(1 × 1) Surface^a^

	*d*_Fe–H_2_O_ (Å)	*d*_Fe–OH_t__ (Å)	*d*_Fe–H_2_O_^iso^ (Å)	*d*_Fe–OHt_^iso^ (Å)
HSE 12%	2.08	1.94	2.12	1.88
HSE 25%	2.07	1.93	2.11	1.88
OptB88-DF	2.07	1.95	2.10	1.90

a The cooperative binding trend is seen in all DFT functionals. The Fe–H_2_O bond lengths are shorter in the dimer when compared to those in the isolated species and vice versa for OH_t_.

**Figure 5:**
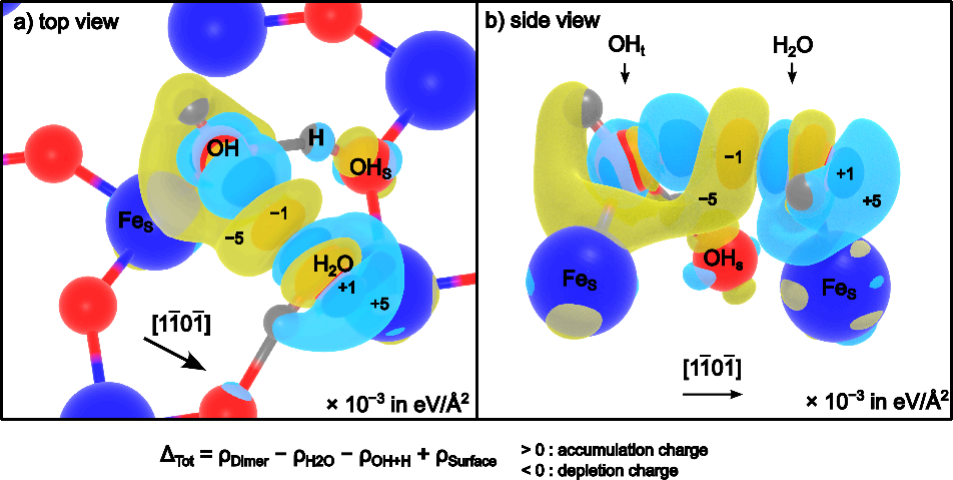
Total variation of charge transfer (Δ_Tot_). a) Top view of the α-Fe_2_O_3_(012)-(1 × 1) surface with isosurfaces of the calculated Δ_Tot_ overlaid. In short, yellow depicts a reduction of charge and cyan depicts an increase in charge when going from isolated OH_t_ and H_2_O species to a H_2_O–OH_t_ dimer. The numbers quantify this change, with values of Δ_Tot_ × 10^–3^ eV/Å^2^ (eq 6). Charge is found to be redistributed to the Fe–H_2_O bond and away from the Fe–OH_t_ bond, which explains the experimentally observed shortening and lengthening of these bonds, respectively. b) A side view of the surface. Red spheres are O atoms, and blue spheres are Fe atoms.

Figure 5 shows isodensities of the calculated Δ_Tot_, which represents the change in charge between isolated H_2_O and OH_t_ + OH_s_ vs the H_2_O–OH_t_ dimer. Yellow isosurfaces depict a reduction in charge in the dimer case when compared to isolated species. Cyan isosurfaces depict an increase in the charge in the dimer case. In general, charge is found to be reorganized away from the Fe–OH_t_ bond and toward the Fe–H_2_O bond via a hydrogen bond in the dimer. This explains the observed changes in the Fe–O bond lengths.

## Discussion

Based on our previous study of the H_2_O/α-Fe_2_O_3_(012)-(1 × 1) system,^20^ H_2_O exposure at 300 K should result in a (1 × 3) overlayer consisting of partially dissociated water dimers (H_2_O–OH_t_). The experimental evidence for this was threefold: (1) temperature-programmed desorption (TPD) data showed a desorption peak at 345 K containing 1.3 D_2_O/unit cell, (2) XPS data showed that approximately half the molecules were dissociated, and (3) nc-AFM images showed 4 protrusions spread across 3 surface unit cells above the positions of the surface Fe cations.^20^ The bimodal apparent height of the protrusions in nc-AFM was reproduced in simulations based on the DFT-determined partially dissociated water dimer structure.^20^ It is important to note, however, that nc-AFM cannot be used to directly retrieve structural information about the species.

The primary result of this NIXSW study is that the oxygen atoms within the intact H_2_O and OH_t_ species have essentially identical adsorption heights above the α-Fe_2_O_3_(012) surface, within experimental error (*H*_H_2_O_ = 1.45 ± 0.04 Å and *H*_OH_t__ = 1.47 ± 0.02 Å). Similar adsorption heights imply similar Fe–O bond lengths as all surface cation sites are equivalent within the bulk truncated Fe_2_O_3_(012) structure. Typically, NIXSW results cannot be trivially converted into a useful measure of bond length as the technique projects the positions onto the bulk crystal unit cell and is blind to any surface relaxations. Here, our DFT calculations show negligible vertical relaxation of the surface of O and Fe for the surface after H_2_O exposure (Figure 1 and Table 1). Assuming bulk continuation lateral positions as found in the nc-AFM images, the measured NIXSW heights give Fe–O bond lengths for both the H_2_O and OH_t_ of approximately 2.0 Å.

This result is in contrast to that of previous quantitative studies of water adsorption on other metal oxides. Normally, metal–O bond lengths are found to be shorter (≤1.9 Å) for OH and longer (≥2.1 Å) for H_2_O.^50,51,54,55^ Reference calculations (HSE 12%) for isolated H_2_O and OH_t_ on α-Fe_2_O_3_(012) indeed yield bond lengths of 2.12 and 1.88 Å, respectively, in line with these expectations (Table 2). However, after the formation of the partially dissociated dimer, the Fe–H_2_O bond length shortens to 2.08 Å while the Fe–OH_t_ bond length extends to 1.94 Å (Table 2). This change can be understood in the context of cooperative binding interactions.^32,33^ In the following, we emphasize the cooperative binding interactions observed in our study following the terminology and work of Schiros et al.^34^ We refer the interested reader to their work for a more complete and detailed explanation.

The central concept in cooperative binding interactions for adsorbed molecules is the balance between surface bonding (S-bonding) and hydrogen bonding (H-bonding). S-bonds and H-bonds can be either acceptor and/or donor bonds in this context, and their equilibrium is crucial for the stability of moieties such as the H_2_O–OH dimer observed here. In short, the stronger the donor bonds received by a species, the stronger the acceptor bonds for that species will be.

For example, an adsorbed H_2_O molecule forms an S-bond with its adjacent surface Fe atom. From the H_2_O molecule’s perspective this bond acts as an acceptor. In its isolated state, a H_2_O molecule does not form H-bonds via its H atom, leading to the absence of donor bonds. If the molecule is instead binding to an adjacent OH molecule it will also form a donor H-bond via its H atom. To maintain balance, the strength of both bonds at the H_2_O molecule will scale with each other; i.e., the existence or an increased strength of the donor H-bond leads to the strengthening of the acceptor S-bond. This phenomenon is cooperative binding, where energy is gained not only by the existence of the H-bond itself but also by its influence on the S-bond.

In the case of the H_2_O in the H_2_O–OH dimer on the α-Fe_2_O_3_(012) surface, the presence of a neighboring OH molecule and the resulting H-bond therefore lead to a strengthening of the Fe–H_2_O surface bond, resulting in a shorter bond length compared to the isolated H_2_O case. This is exactly what we observe experimentally. This enhancement is also confirmed by charge accumulation in the Fe–H_2_O binding area in Figure 5, indicating improved and stronger hybridization with the Fe atom, again compared to the isolated case.

As with an isolated H_2_O, an isolated OH also forms an acceptor S-bond with its adjacent Fe surface atom and lacks any donor bonds. However, if it forms a hydrogen bond with a neighboring H_2_O, from its perspective, it acquires a second acceptor bond. In contrast to the H_2_O case, the two acceptor bonds end up competing with each other to maintain balance. Thus, in the case of OH in the H_2_O–OH dimer, the Fe–OH surface bond is weakened from this competition. This is evident as a longer bond as we observe and a charge depletion in the Fe–OH area (Figure 5).

As well as the α-Fe_2_O_3_(012) surface, similar cooperative binding arguments have been invoked to explain the formation of partially dissociated dimers on other surfaces such as Fe_3_O_4_(001),^22^ Fe_3_O_4_(111),^23,24^ RuO_2_(110),^25^ Mg(100),^26^ PdO(101),^27^ and ZnO(1010).^28^ Scanning probe microscopy, temperature-programmed desorption (TPD), and DFT were used to investigate the preferential formation of H_2_O–OH dimers over isolated species, with these prior studies all concluding that such a phenomenon could be explained by the formation of favorable hydrogen bonds between the adsorbed species. For example, Haywood et al. undertook a TPD/DFT study of H_2_O on PdO(101)^27^ and came to the same conclusions; an isolated OH species has restricted H-bonding on PdO(101), so H_2_O–OH dimers with a favorable balance of H-bonding and S-bonding are required for dissociation. In the end, our results provide the first quantitative and direct structural evidence of this hydrogen bonding effect in the observed dimers.

## Conclusions

We have shown that the H_2_O and OH_t_ of the (1 × 3) overlayer on α-Fe_2_O_3_(012)-(1 × 1) sit close to bulk continuation adsorption heights (*H*_H_2_O_ = 1.45 ± 0.04 Å and *H*_OH_t__ = 1.47 ± 0.02 Å), corroborating our prior nc-AFM/DFT study.^20^ We have also discerned the adsorption height of the OH_s_ species located in the surface (*H*_OH_s__ = −0.01 ± 0.02 Å), which is essentially in plane with the surface oxygen atoms.

The H_2_O and OH_t_ both sit essentially coplanar with similar Fe–O bond lengths (∼2.0 Å). This stands in contrast to prior studies of isolated H_2_O and OH on other metal oxides.^50,51,54,55^ Typically, on other surfaces, the H_2_O bond length is found to be longer and the OH shorter than what was found in this study. We explain these unexpected Fe–O bond lengths by the formation of a hydrogen bond between H_2_O and OH_t_. In turn, this hydrogen bond affects the strengths of the Fe–O bonds via charge reorganization.^34^ This is the first direct and quantitative measure of this cooperative binding effect, which was enabled by the formation of isolated H_2_O–OH dimers on the α-Fe_2_O_3_(012)-(1 × 1) surface.

More broadly, these results emphasize the importance of considering H_2_O–OH interactions on metal oxide surfaces. As seen, these interactions play a central role in defining the dissociative behavior of H_2_O, which is an important phenomenon for applications of metal oxides in catalysis. It is also a central phenomenon for informing the acid/base behavior of metal oxide surfaces, which is important in general mineralogy.

## Supporting Information Available

Further DFT details, calculations of the OH positions, comparison of all DFT functionals, C 1s SXPS spectra (PDF)
