# Supplementary material for: Quantitative Measurement of Cooperative Binding in Partially Dissociated Water Dimers at the Hematite “R-Cut” Surface
Source: J Phys Chem C Nanomater Interfaces. 2024 Sep 30;128(40):16977–85. doi: 10.1021/acs.jpcc.4c04537 (PMC11481491; doi:10.1021/acs.jpcc.4c04537)
Supplement: Supplementary file 1 — jp4c04537_si_001.pdf [file jp4c04537_si_001.pdf]

Supplementary Information

Quantitative Measurement of Cooperative Binding In Partially Dissociated Water Dimers at the Hematite "R-Cut" Surface

Paul T. P. Ryan[1], Panukorn Sombut[1], Ali Rafsanjani-Abbasi[1], Chunlei Wang[1], Fulden Eratam[2], Francesco Goto[2,3], Cesare Franchini[4], Ulrike Diebold[1], Matthias Meier[1,4], David A. Duncan[2], Gareth S. Parkinson[1*]

[1]Institute of Applied Physics, Technische Universität Wien, 1040, Vienna, Austria

[2]Diamond Light Source, Harwell Science and Innovation Campus, OX11 0QX, Didcot, UK

[3]Politecnico di Milano, Piazza Leonardo da Vinci, 20133 Milano MI, Italy

[4]Faculty of Physics and Center for Computational Materials Science, Uni. of Vienna, 1040, Vienna, Austria

*parkinson@iap.tuwien.ac.at (phone: +43(0)664605883433)

Contents

1. **DFT further details** 1

2. **NIXSW OH position calculation** 2

Defining the models 2

Calculating p_{OHt} and p_{OHs} 3

Discussion of models 3

Model 1 3

Model 2 4

Model 3 and 4 4

Model 5 and 6 4

3. **Comparison of all DFT functionals** 4

Optimized vs experimental lattice parameters 4

Comparison of functionals 9

Observation of cooperativity in DFT 11

4. **C 1s photoemission spectra** 12

References 13

1. DFT further details

All the calculations were performed using the Vienna *ab initio* Simulation Package (VASP).[1,2] The Projector Augmented Wave (PAW) approach[3,4] was used for handling the near-core regions, with a basis set cut-off energy of 550 eV. Calculations were initially performed using the Perdew-Burke-Ernzerhof (PBE)[5] and revised PBE (revPBE)[6] exchange-correlation functional with an effective on-site Coulomb repulsion term U_{eff} = 5.0 eV[7] for Fe atoms to model the oxide. Calculations are spin-polarized and performed at the Γ-point only for the (2 × 3) supercells and a Γ-centered k-mesh of 6×1×3 for the bulk optimization. Convergence is achieved when an electronic energy step of 10^{-6} eV is obtained, and forces acting on ions smaller than 0.02 eV/Å. Different van der Waals implementations were tested such as vdW corrections according to the method of Grimme et al. (DFT-D2)[8] with zero-damping (DFT-D3)[9]

and the correction proposed by Dion *et al*. and Klimes *et al*. (vdW-DF, vdW-DF2, optPBE-DF, optB88-DF and optB86b-DF)[10–12]. We also utilized metaGGA functional (SCAN and R2SCAN) with the inclusion of vdW (rVV10)[13–16] and an on-site Coulomb repulsion term U_{eff} = 3.10 eV.[17] We also tested a variant optimized specifically for solids, PBEsol,[18] which accounts for the well-known issue of disfavored density overlapping present in PBE, using the same U_{eff}. The hybrid functional (HSE06)[19] was investigated with the standard mixing factor 12% and 25% and screening length (0.2^{-1} $Å^{-1}$). The supercell was reduced to (1 × 3) supercell with k-meshes 3×1×1. Symmetric slabs were built, consisting of four Fe_4O_6 layers in thickness where only the two inner central O layers are kept fixed. The bottom surface is saturated with a full monolayer of water molecules and left untouched throughout the study. A vacuum region of 15 Å between consecutive slabs normal to the surface is added to avoid interactions.

2. NIXSW OH position calculation

Defining the models

To calculate the individual OH_t (OH species bound to a surface cation) and OH_s (OH species formed from a lattice oxygen atom on the surface) coherent positions from the single NIXSW measurement of the O_{OH} photoemission peak, a model of the OH distribution must be defined based on a set of assumptions about the possible distributions the OH species can take. Here we will outline all the models tested in this study including the model that is used in the main article.

All models are a two-atom model with each atom in the model representing a coherent location of one of the OH species (OH_t and OH_s). In such a case, individual coherent positions, p, and coherent fractions, f, for each atom can be defined (p_{OH_t}, p_{OH_s} and f_{OH_t}, f_{OH_s}). Note that for clarity the hkl subscript denoting the reflection has been omitted and replaced with the species type. The coherent positions (p_{OH_t} and p_{OH_s}) are unknown and to be determined. The coherent fractions (f_{OH_t} and f_{OH_s}) are defined by the specific model and are the relative populations of the two sites. These coherent fractions must sum to unity: $f_{OH_t} + f_{OH_s} = 1$.

The measured coherent fraction, f_m, may be less than unity due to a specific distribution of atoms (as to be determined) but also due to molecular and crystal vibrations and contributions from decoherent species. These considerations lead to the following definition of f_m:

$$f_m = f_{str} \cdot D_H \cdot C \tag{1}$$

where f_{str} is the true structural coherent fraction due to the specific distribution of the two atoms alone.[20] D_H is the Debye-Waller factor which defines how much the coherent fraction is reduced due to molecular and crystal vibrations. This kind of vibrational data is rare for specific species on specific surfaces. So for this study, D_H of the OH species will take only two values; $D_H = 1.0$ i.e. no vibration or $D_H = 0.91$ which is the Debye-Waller factor measured in this study for H_2O. C is an order parameter which defines the fraction of OH species that coherently contributes to the signal. For example, for the model used in the main article $C = 0.79$ from considering that all extra 21% OH contributes incoherently.

Six models with differing values of D_H, C and f_{OH_t}:f_{OH_s} ratio have been investigated. p_{OH_t} and p_{OH_s} for each of the models were calculated along with heights, H_{OH_t} and H_{OH_s}, above a bulk truncated (012) oxygen surface. H_{OH_t} and H_{OH_s} were calculated according to equation 1 in the main article with values of $n = 1$ and $n = 0$ respectively. These results are presented in Table S1 along with the parameters defined. The next section outlines the details of the calculations while the final section discusses these results.

Table S1 – Results of all models tested in this study with model 2 being the model used in the main article. H_{OH_t} *and* H_{OH_s} have been calculated using equation 1 from the main article with $n = 1$ and $n = 0$ respectively. The errors in the H_{OH_t} *and* H_{OH_s} values are all $\pm$ 0.02 Å.

Model #	f_{OH_t}:f_{OH_s}	D_H	C	H_{OH_t} (Å)	H_{OH_s} (Å)
1	0.5:0.5	1	1	1.38	0.09
2 (main article)	0.5:0.5	0.91	0.79	1.47	-0.01
3	0.55:0.45	0.91	0.90	1.46	0.07
4	0.45:0.55	0.91	0.90	1.40	0.01
5	0.8:0.2	0.91	1	1.60	0.50
6	0.2:0.8	0.91	1	0.97	-0.13

Calculating p_{OH_t} and p_{OH_s}

The structural information of each atom and the measurement results can each be represented as a vector in the complex plane (structure factors for the given reflection[20,21]) with values of p and f being the phase and magnitude, respectively, of each vector. The vectors of the two individual atom sites are $Z_{OH_t} = f_{OH_t} e^{2\pi i p_{OH_t}}$ and $Z_{OH_s} = f_{OH_s} e^{2\pi i p_{OH_s}}$ and their sum:

$$Z_m = Z_{OH_t} + Z_{OH_s} = f_{str} e^{2\pi i p_m} \tag{2}$$

produces a third vector, Z_m, who's p_m and f_{str} values are retrieved through the NIXSW measurement. The magnitudes of these vectors can be related to the difference in the phase, Δp, between Z_{OH_t} and Z_{OH_s} as such:

$$|Z_m|^2 = |Z_{OH_t}|^2 + |Z_{OH_s}|^2 + 2|Z_{OH_t}||Z_{OH_s}| \cos(2\pi\Delta p) \tag{3}$$

which can be rearranged and recast with respect to the vector magnitudes, f_{str}, f_{OHt} and f_{OHs}:

$$\cos(2\pi\Delta p) = \frac{f_{str}^2 - f_{OH_t}^2 - f_{OH_s}^2}{2 f_{OH_t} f_{OH_s}} \tag{4}$$

The unit vectors $\hat{Z}_{OH_t}$ and $\hat{Z}_{OH_s}$ can then be defined dependent on Δp and the measurement unit vector $\hat{Z}_m = e^{2\pi i p_m}$:

$$\hat{Z}_{OH_t} = \frac{f_{OH_t}}{f_{str}} e^{2\pi i p_m} + \frac{f_{OH_s}}{f_{str}} e^{2\pi i (p_m - \Delta p)} \tag{5}$$

$$\hat{Z}_{OH_s} = \frac{f_{OH_s}}{f_{str}} e^{2\pi i p_m} + \frac{f_{OH_t}}{f_{str}} e^{2\pi i (p_m + \Delta p)} \tag{6}$$

where the choice of plus or minus sign in $e^{2\pi i (p_m \pm \Delta p)}$ is such that the OH_s has the larger p_{OH_s}. Finally, the individual phases of the vectors, p_{OH_t} and p_{OH_s}, are retrieved with $p = \frac{1}{2\pi} \tan^{-1}\left(\frac{Im(\hat{Z})}{Re(\hat{Z})}\right)$.

Discussion of models

Model 1

Model 1 (Table S1) calculates the heights not taking into account any molecular and crystal vibrations ($D_H = 1$) and assuming that all the OH species contribute coherently via the two atom sites ($C = 1$) by ignoring the excess 21% OH. This model places the OH_t lower than the H_2O ($H_{OH_t} = 1.38 \pm 0.02$ Å vs $H_{H_2O} = 1.45 \pm 0.04$ Å), which is counter to the trend seen in all of the DFT results, though the significance of the difference is minor and typically a 0.1 Å difference between theory and experiment would be considered negligible.

Model 2

Model 2 includes the effect of both molecular and crystal vibrations and the possible decoherent effect of the excess OH. D_H was set $D_H = 0.91$ which is the D_H of H_2O on this surface and C was set $C = 0.79$ from considering that all of the extra 21% OH species incoherently contribute. Including these affects in model 2 increases the separation between p_{OH_t} and p_{OH_s} and moves the OH_t slightly higher than the H_2O as is observed in the DFT calculations. The height of the OH_s is also still reasonable placing it in plane with the surface oxygens. This is the model that has been used in the main article.

Model 3 and 4

Models 3 and 4 place half of the excess OH coherently in either the OH_t or the OH_s sites respectively. This simulates a partial coherent contribution from the defect sites. The other half of the excess OH is considered incoherently contributing via the order parameter $C = 0.90$. In both models, $D_H = 0.91$ as for model 2 and H_2O. The H_{OH_t} in models 3 and 2 are the same within the experimental error. However, model 3 places the OH_s more above the O surface plane (in contrast to model 2) which is consistent with the DFT. It is not unreasonable to think that any excess OH may be due to water adsorption at surface defects which contribute somewhat coherently to the measurement and model 4 could be reflecting this, similar to what is observed for water on $TiO_2(110)$.[22]

Model 5 and 6

Finally, models 5 and 6 place all of the excess OH coherently in the OH_t or the OH_s sites respectively ($C = 1$ and $D_H = 1$ in both cases). These models are unlikely, given the prior AFM/ XPS/ DFT study and are provided here as extreme cases. These models either place the OH_s physically too high ($H_{OHs} = 0.50 \pm 0.02$ Å for model 5) or the OH_t too low ($H_{OH_t} = 0.97 \pm 0.02$ Å for model 6) and are thus unlikely. Models with higher relative populations at either site (> 0.8) are incalculable if D_H and C are to remain fairly high (i.e. $D_H \geq 0.9$ and $C \geq 0.79$). Though $D_H < 0.9$ would require unreasonably large molecular and crystal vibrational amplitudes and $C < 0.79$ would require the observation of more defects.

3. Comparison of all DFT functionals

Optimized vs experimental lattice parameters

Table S*2* shows the DFT results for all functionals tested using the experimental lattice parameters (a = 5.038 Å and c = 13.77 Å). However, bulk structure calculations can be carried out for each functional and so called optimized lattice parameters can be retrieved from the resulting relaxed bulk structure. Generally, these optimized parameters are different to the experimental lattice parameters. This is depicted in Figure S1 which shows for each functional the difference between the optimized and experimental a (Figure S1 a) and c (Figures S1 b) lattice parameters. Generally speaking, the hybrid calculations perform the best in reproducing the experimental lattice parameters, the strongest deviations from experiment are found for functionals with dispersion corrections and the SCAN functionals all underpredict the lattice parameters.

The question arises as to which set of lattice parameters to use; optimized vs experimental. As such, a second set of calculations of the surface were undertaken but instead using separate lattice parameters from optimized bulk structure calculations for each functional. These results are provided in Table S3. However, the direct comparison of the absolute heights between the optimized and experimental lattice parameters is not possible. This is because changing the lattice parameters changes the reflection layer spacing (d_{hkl}) rendering any direct comparison of the absolute DFT heights with the NIXSW heights impossible. Moreover, a deviation from the experimental lattice parameters represents a relaxation of the structure and this would work to change the projected height of adsorbed species while not necessarily changing the height with respect to the oxygen surface layer. As such, instead of the absolute heights, the coherent positions, p_{024}, must be compared between optimized and experimental lattice

parameters. In this way, the coherent positions are normalized heights with respect to d_{hkl} and this therefore removes the effects of any changes to d_{hkl}.

Figure S2 shows a comparison between the p_{024} of the optimized and experimental lattice parameter for the H_2O molecule in the H_2O-OH_t dimer. When using the experimental lattice parameters, a number of functionals give values close to the NIXSW $p_{024} = 0.79$ value (with respect to a projected bulk oxygen surface layer at $p_{024} = 0.61$). The best performing are the PBE, PBE-D3, optB88-DF and HSE 12% (Table S2). However, when using the optimized lattice parameters, all the functionals over bind the position of the H_2O with none being able to reproduce the NIXSW $p_{024} = 0.79$ value. The highest reached p_{024} are for the revPBE and vdw-DF functionals ($p_{024} = 0.76$ and 0.77) though this is likely due to their very large overprediction of the lattice parameters ($\Delta c = +0.18$ Å and $+0.23$ Å).

The Fe-O bond lengths for the H_2O and OH_t species will also affect the calculated heights. Figure S3 shows the bond lengths for the H_2O ($d_{Fe\text{-}H2O}$) and OH_t ($d_{Fe\text{-}OHt}$) species when calculated using the optimized (Figure S3 a and b) and experimental (Figure S3 c and d) lattice parameters. The bond lengths are essentially the same in each case and both follow the same trend as in Figure S1 for the deviation from experiment. This makes clear that there are negligible differences in local effects between the optimized and experimental lattice parameters and any differences are almost entirely from bulk and/or surface relaxations.

To further quantify the correlation of the bulk and surface structural relaxations with the relevant structural values (e.g. heights and bond lengths), correlation coefficients were calculated for all parameters in Tables S1 and S2 with respect to Δc as well as for the height difference, ΔH, between the H_2O and OH_t species. Table S3 provides these coefficients. In both the optimized and experimental lattice parameter cases, all heights and bond lengths show extremely strong correlation with Δc. Slightly weaker correlation is found for Fe-OH bond lengths (coefficient > 0.6) compared to Fe-H_2O bond lengths (coefficient > 0.9) and this is likely due to the more ionic character of the Fe-OH bond. For the optimized case, the OH_s height shows no correlation with Δc (coefficient = 0.04) but this can be attributed to the OH_s sitting at a bulk position who's coherent position would be consistent. The only parameter showing little or no correlation with Δc for both the optimized and experimental lattice parameter cases is ΔH. ΔH can be thought of as a measure of the cooperativity effect. This lack of correlation is this likely due to differences in local effects defining the strength of the cooperativity between each functional.

In light of all of the above, the experimental lattice parameters are used for comparison of the DFT to the NIXSW results and will be used throughout.

Table S2 – **DFT calculations using experimental lattice parameters (a = 5.038 Å, c = 13.77 Å).** The DFT heights (H_{H2O}, H_{OHt} and H_{OHs}), with respect to an oxygen bulk terminated (012) surface for each functional with comparison to the NIXSW results. Values in brackets for the NIXSW results are the error in the last significant figure. d_{Fe-H2O} and d_{Fe-OHt} are the relevant bonds lengths to surface Fe cations. $d_{Fe-H2O(iso)}$ and $d_{Fe-OHt(iso)}$ are the bond lengths for isolated species. p_{024}^{H2O} is the coherent position of the H_2O. Δc is the difference between the experimental and calculated c unit cell parameter for α-Fe_2O_3. The green highlighted rows show the results presented in the main article.

Functional	U_{eff} (eV)	H_{H2O} (Å)	H_{OH_t} (Å)	H_{OH_s} (Å)	$d_{\text{Fe-H2O}}$ (Å)	$d_{\text{Fe-OHt}}$ (Å)	$d_{\text{Fe-H2O(iso)}}$ (Å)	$d_{\text{Fe-OHt(iso)}}$ (Å)	p_{024}^{H2O}	Δc (Å)
NIXSW	n/a	1.45(4)	1.47(2)	-0.01(2)	n/a	n/a	n/a	n/a	n/a	n/a
PBE	5	1.47	1.52	0.16	2.07	1.96	2.11	1.90	0.80	0.08
PBE-D2	5	1.38	1.45	0.10	2.06	1.96	2.10	1.90	0.75	-0.10
PBE-D3	5	1.43	1.49	0.13	2.07	1.95	2.11	1.90	0.78	0.03
vdw-DF	5	1.69	1.75	0.32	2.13	1.96	2.16	1.91	0.92	0.23
vdW-DF2	5	1.77	1.84	0.40	2.12	1.96	2.15	1.91	0.96	0.31
optPBE-DF	5	1.52	1.57	0.18	2.09	1.96	2.12	1.90	0.83	0.10
optB88-DF	5	1.42	1.47	0.11	2.07	1.95	2.10	1.90	0.77	0.02
optB86b-DF	5	1.38	1.42	0.07	2.06	1.95	2.09	1.90	0.75	-0.02
SCAN	5	1.30	1.35	0.00	2.04	1.94	2.07	1.88	0.70	-0.10
SCAN+rVV10	5	1.28	1.34	0.01	2.03	1.93	2.07	1.89	0.69	-0.12
R2SCAN	5	1.32	1.37	0.03	2.04	1.94	2.09	1.89	0.72	-0.07
R2SCAN+rVV10	5	1.29	1.34	0.00	2.04	1.94	2.08	1.89	0.70	-0.11
revPBE	5	1.63	1.68	0.27	2.11	1.96	2.15	1.91	0.88	0.18
PBEsol	5	1.27	1.32	0.00	2.03	1.95	2.06	1.90	0.69	-0.10
HSE06(25%)	n/a	1.36	1.44	0.06	2.07	1.93	2.11	1.88	0.74	-0.01
HSE06(12%)	n/a	1.42	1.50	0.11	2.08	1.94	2.12	1.88	0.77	0.06
SCAN	3.1	1.30	1.35	0.01	2.04	1.94	2.08	1.88	0.71	-0.06
SCAN+rVV10	3.1	1.28	1.34	0.01	2.04	1.94	2.08	1.88	0.70	-0.09
R2SCAN	3.1	1.34	1.41	0.05	2.05	1.94	2.09	1.89	0.73	-0.03
R2SCAN+rVV10	3.1	1.30	1.35	0.01	2.05	1.94	2.08	1.89	0.71	-0.06

Table S3 - **DFT calculations using optimized lattice parameters**. The DFT heights (H_{H2O}, H_{OHt} and H_{OHs}), with respect to an oxygen bulk terminated (012) surface for each functional with comparison to the NIXSW results. Values in brackets for the NIXSW results are the error in the last significant figure. d_{Fe-H2O} and d_{Fe-OHt} are the relevant bonds lengths to surface Fe cations. $d_{Fe-H2O(iso)}$ and $d_{Fe-OHt(iso)}$ are the bond lengths for isolated species. p_{024}^{H2O} is the coherent position of the H_2O. Δc is the difference between the experimental and calculated c unit cell parameter for α-Fe_2O_3.

Functional	U_{eff} (eV)	H_{H2O} (Å)	H_{OHt} (Å)	H_{OHs} (Å)	$d_{\mathrm{Fe-H2O}}$ (Å)	$d_{\mathrm{Fe-OHt}}$ (Å)	$d_{\mathrm{Fe-H2O(iso)}}$ (Å)	$d_{\mathrm{Fe-OHt(iso)}}$ (Å)	p_{024}^{H2O}	Δc (Å)
NIXSW	n/a	1.45(4)	1.47(2)	-0.01(2)	n/a	n/a	n/a	n/a	0.79	n/a
PBE	5	1.38	1.43	0.08	2.08	1.96	2.11	1.90	0.75	0.08
PBE-D2		1.36	1.43	0.09	2.06	1.95	2.10	1.90	0.74	-0.10
PBE-D3		1.38	1.44	0.08	2.07	1.96	2.11	1.90	0.75	0.03
vdw-DF		1.44	1.48	0.07	2.13	1.96	2.17	1.91	0.77	0.23
vdW-DF2		1.43	1.48	0.07	2.13	1.96	2.17	1.91	0.76	0.31
optPBE-DF		1.40	1.45	0.07	2.09	1.96	2.13	1.90	0.75	0.10
optB88-DF		1.37	1.42	0.06	2.07	1.95	2.10	1.90	0.74	0.02
optB86b-DF		1.36	1.41	0.06	2.06	1.95	2.09	1.90	0.74	-0.02
SCAN		1.36	1.40	0.06	2.03	1.94	2.07	1.89	0.74	-0.10
SCAN+rVV10		1.34	1.39	0.06	2.03	1.94	2.06	1.89	0.73	-0.12
R2SCAN		1.36	1.40	0.06	2.04	1.94	2.08	1.89	0.74	-0.07
R2SCAN+rVV10		1.35	1.40	0.06	2.04	1.94	2.08	1.89	0.74	-0.11
revPBE		1.42	1.47	0.07	2.12	1.96	2.16	1.90	0.76	0.18
PBEsol		1.34	1.39	0.07	2.03	1.95	2.06	1.90	0.73	-0.10
HSE06(25%)	n/a	1.36	1.44	0.06	2.07	1.93	2.11	1.88	0.74	-0.01
HSE06(12%)		1.37	1.46	0.07	2.08	1.94	2.12	1.88	0.74	0.06
SCAN	3.1	1.36	1.42	0.07	2.04	1.94	2.07	1.89	0.74	-0.06
SCAN+rVV10		1.36	1.42	0.07	2.04	1.94	2.07	1.89	0.74	-0.09
R2SCAN		1.36	1.42	0.07	2.05	1.94	2.09	1.89	0.74	-0.03
R2SCAN+rVV10		1.36	1.42	0.07	2.05	1.94	2.08	1.89	0.74	-0.06

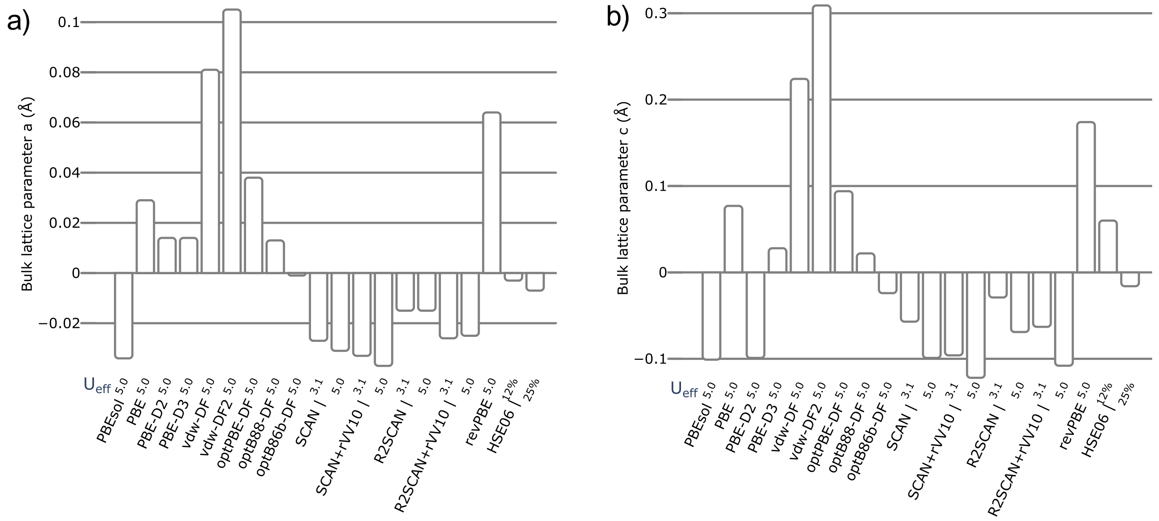

Figure S1 – The difference between the experimental a) bulk a lattice parameter and b) bulk c lattice parameter with that of an optimized bulk structure calculation for each functional.

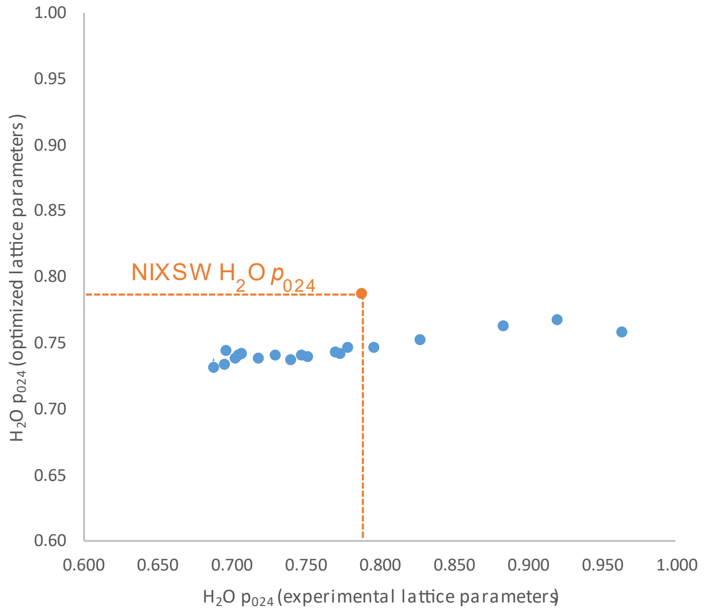

Figure S2 – The coherent positions, p_{024}, of the H_2O for calculations using the optimized vs experimental lattice parameters. Only when using the experimental lattice parameters can the DFT reproduce the NIXSW structural results. And only those functionals that have small bulk or surface relaxations are suitable for the structure calculations.

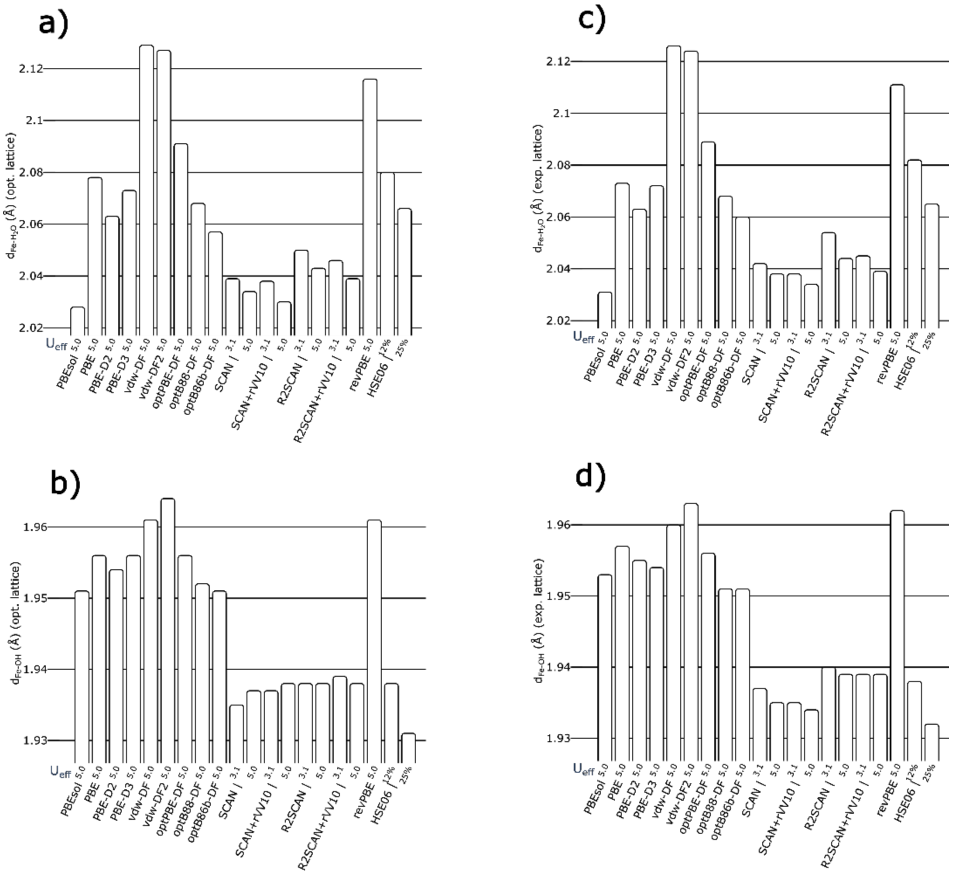

Figure S3 – The Fe-O bond lengths for the H_2O and OH_t species using the optimized (a and b) and experimental (c and d) lattice parameters. The trends across the functionals are identical between optimized vs experimental and correlate with Figure S1.

Table S3 – Calculated correlation coefficients for Δc with respect to the heights (H), bond lengths (d), H_2O coherent positions (p_{024}^{H2O}) and differences between the H_2O and OH_t heights (ΔH). Both optimized and experimental lattice parameter cases are provided. For all heights and bond lengths there is extremely strong correlation in both cases. For ΔH there is very little correlation for either the optimised or experimental cases. This is likely due to differences in local effects defining the strength of the cooperativity between each functional.

	H_{H2O}	H_{OHt}	H_{OHs}	$d_{\mathrm{Fe-H2O}}$	$d_{\mathrm{Fe-OHt}}$	$d_{\mathrm{Fe-H2O(iso)}}$	$d_{\mathrm{Fe-OHt(iso)}}$	p_{024}^{H2O}	ΔH
optimised	0.94	0.91	0.04	0.96	0.73	0.96	0.60	0.88	-0.15
experimental	0.98	0.98	0.97	0.96	0.72	0.95	0.62	0.98	0.17

Comparison of functionals

Figure S4 shows the difference between the DFT and NIXSW heights for each functional using the experimental lattice parameters for the H_2O, OH_t and OH_s species. Unsurprisingly, across all functionals there is a strong correlation with Figure S1, as the bulk and/or surface relaxations will directly affect the calculated heights and there is a direct correlation of this with the Fe-O bond lengths (Figures S3 and Table S3). Taking into account all three species, the best performing functionals are: PBE, PBE-D3 optB88-DF and HSE12%.

While the PBE functional closely reproduces the NIXSW heights, this is likely a coincidence due to a strong relaxation of the unit cell outward, normal to the surface. This is evident from the large deviation of its c lattice parameter ($\Delta c = +0.08$ Å). As such, the best performing functionals which take into

account bulk and surface relaxations are the PBE-D3, optB88-DF and HSE 12% calculations as these all have very low deviations from the experimental lattice parameters.

While reproducing the absolute values of the NIXSW results, the best performing functional should also reproduce observed experimental trends. It is clear that the NIXSW results demonstrate that the H_2O and OH_t sit very close in height to one another and this can generally be thought of as a direct consequence of the cooperativity effect. More specifically, all reasonable distribution Models for the NIXSW results place the OH_t at essentially the same height as the H_2O in the range -0.07 – +0.02 Å.

Figure S5 shows the combined relative differences between the H_2O and OH_t heights for each functional using the experimental lattice parameters. It is clear from Figure S4 that the PBE-D3 and hybrid calculations perform poorly, consistently placing the OH_t much too high compared to the H_2O (+ 0.08 Å). The best functional in this regard is optB88-DF which places the H_2O and OH_t close in height (+ 0.04 Å).

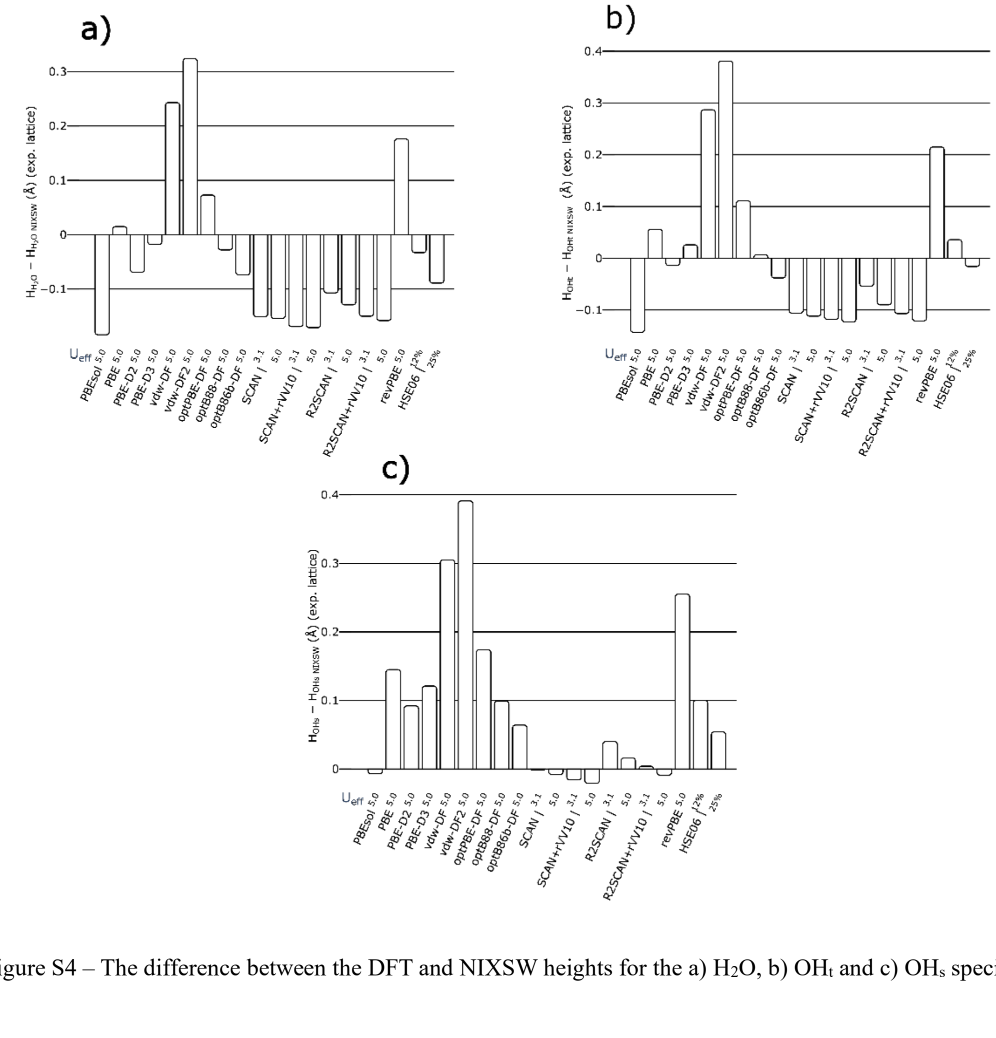

Figure S4 – The difference between the DFT and NIXSW heights for the a) H_2O, b) OH_t and c) OH_s species.

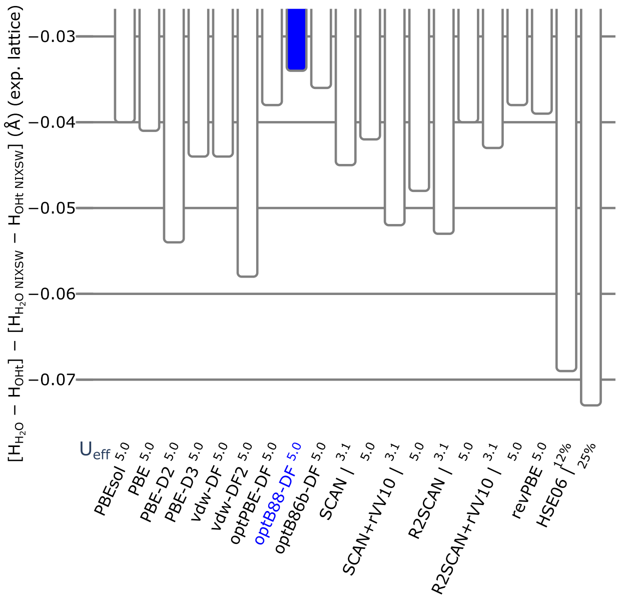

Figure S5 – The combined relative height differences between the H_2O and OH_t species. Marked in blue is the optB88-DF functional, which in this regards performs the best, placing the H_2O and OH_t species very close in height.

Observation of cooperativity in DFT

Figure S6 shows the difference between the bond lengths of H_2O and OH_t in the H_2O-OH_t dimer vs for the isolated species (using experimental lattice parameters). This demonstrates directly the strength of the cooperativity effect. All functionals show some amount of cooperativity, with the H_2O bond being shorter and the OH_t bond being longer in the dimer in all cases. However, there are large differences in the strength of the cooperativity between the functionals. While the optB88-DF performs well in reproducing the NIXSW results, it gives a fairly mediocre cooperativity effect. Stronger cooperativity is seen in the hydrid and revPBE calculations. This shows that, while the cooperativity effect plays a role in the similar measured adsorption heights of the H_2O and the OH_t, other effects, such as specific surface atom relaxation, would also play a role. Though such effects become harder to experimentally pinpoint.

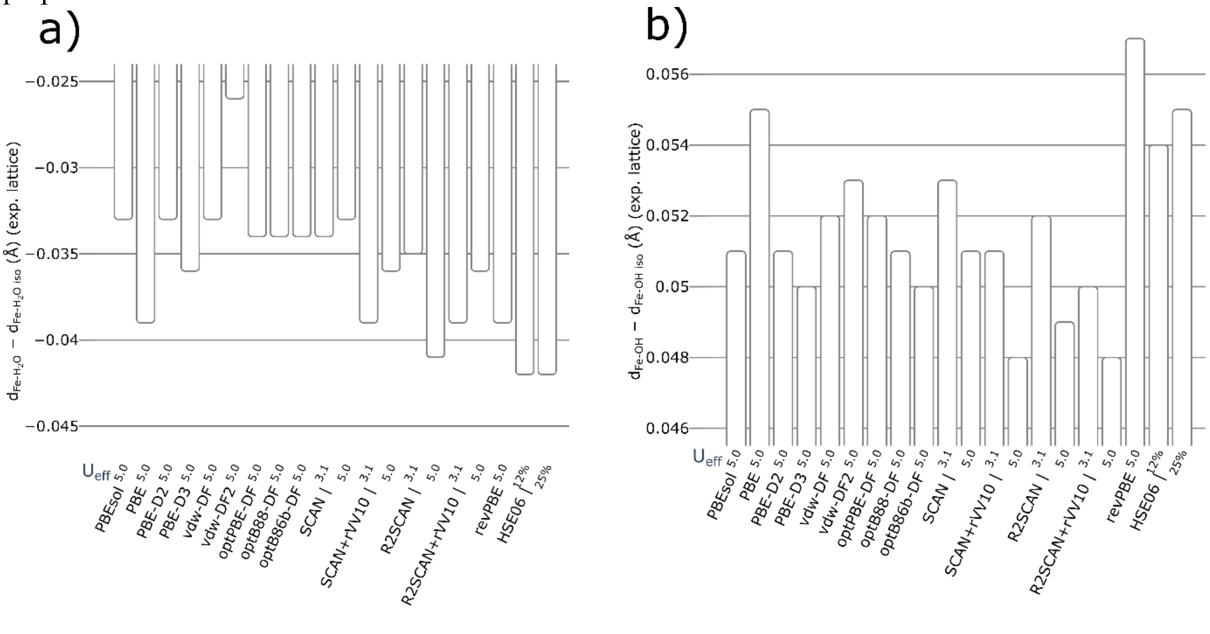

Figure S6 – The difference between the Fe-O bond lengths for isolated molecules vs molecules found in the H_2O-OH_t dimer for the a) H_2O and b) OH_t species. These DFT calculations utilized the experimental lattice parameters. This figure directly depicts the cooperativity effect. All functionals show a cooperativity effect.

4. C 1s photoemission spectra

Figure S7 shows the C 1s spectra before and after H_2O exposure to the Fe_2O_3(012) surface with comparison to the O 1s spectra given in the main article. It is seen that there is some very slight carbon contamination before H_2O deposition with only a small increase in an oxidized carbon peak (288.5 eV) after H_2O deposition (Fig. S7c). There is also some potassium present in the sample which is not surprising given the fact that the crystals are natural hematite and such alkali metal contaminants are readily observed in the bulk of such crystals.

The oxidized carbon peaks (288.5 eV and 285.5 eV) are likely from C=O double bonds and C-O single bonds respectively. These could contribute to the OH photoemission peak intensity, in particular, to the extra OH photoemission intensity observed. We calculate that the C 1s oxidized peak intensity at 285.5 eV would only account to 30 % of the extra OH photoemission intensity. Thus, it is likely that this extra OH photoemission intensity comes from additional sources as outlined in the main article. Moreover, models 3 and 4 presented in Section 2 of this SI show via the order parameter *C* that any coherent contribution of the extra OH intensity has minimal effect on the final conclusions of the study.

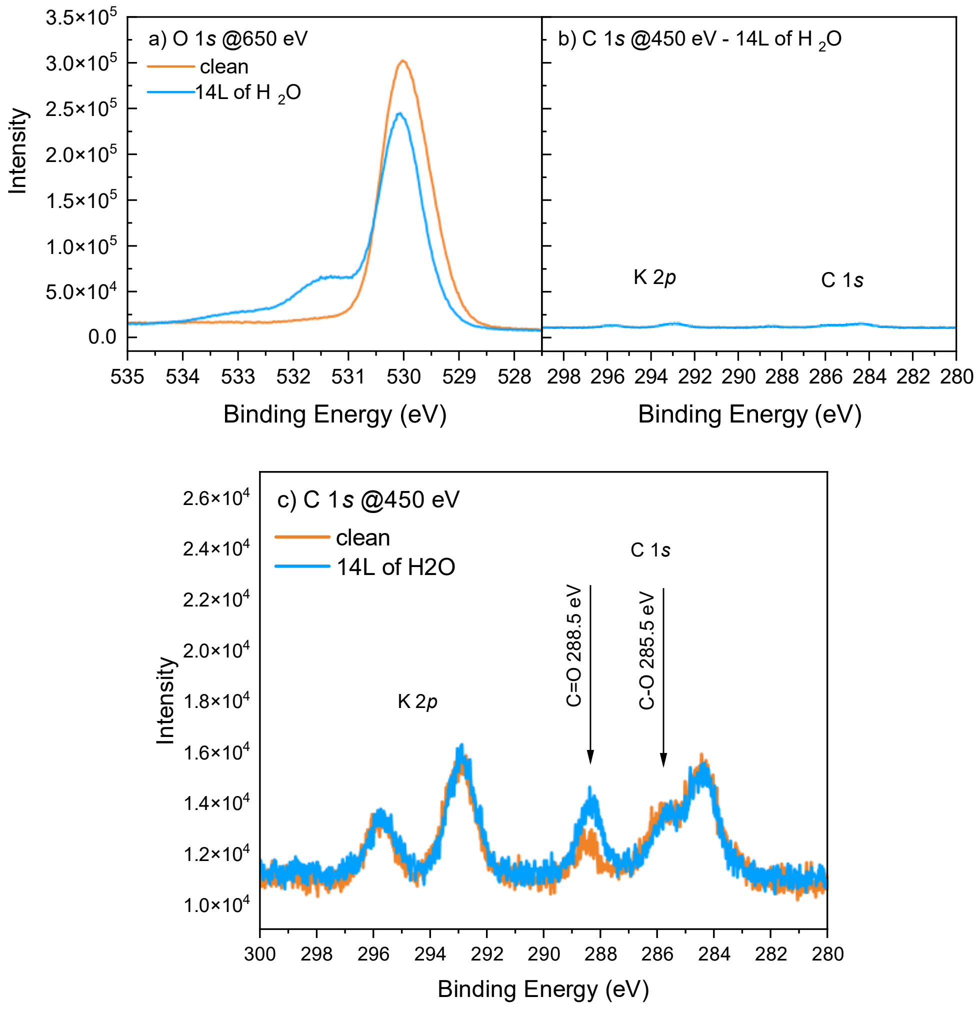

Figure S7 – C 1s and O 1s spectra of the surface both before and after H_2O exposure. a) The O 1s spectra from the main article. b) The C 1s spectrum after H_2O exposure at the same intensity scale as the O 1s spectra. c) The same C 1s spectrum but a more appropriate scale and with comparison to before H_2O exposure. There is some negligible C and K contamination.

References

(1) Kresse, G.; Furthmüller, J. Efficiency of Ab-Initio Total Energy Calculations for Metals and Semiconductors Using a Plane-Wave Basis Set. *Comput Mater Sci* **1996**, *6* (1), 15–50. https://doi.org/10.1016/0927-0256(96)00008-0.

(2) Kresse, G.; Hafner, J. Ab Initio Molecular Dynamics for Open-Shell Transition Metals. *Phys Rev B* **1993**, *48* (17), 13115–13118. https://doi.org/10.1103/PhysRevB.48.13115.

(3) Kresse, G.; Joubert, D. From Ultrasoft Pseudopotentials to the Projector Augmented-Wave Method. *Phys Rev B Condens Matter Mater Phys* **1999**, *59* (3), 1758–1775. https://doi.org/10.1103/PhysRevB.59.1758.

(4) Blöchl, P. E. Projector Augmented-Wave Method. *Phys Rev B* **1994**, *50* (24), 17953–17979. https://doi.org/10.1103/PhysRevB.50.17953.

(5) Perdew, J. P.; Burke, K.; Ernzerhof, M. Generalized Gradient Approximation Made Simple. *Phys Rev Lett* **1996**, *77* (18), 3865–3868. https://doi.org/10.1103/PhysRevLett.77.3865.

(6) Zhang, Y.; Yang, W. Comment on "Generalized Gradient Approximation Made Simple." *Phys Rev Lett* **1998**, *80* (4), 890. https://doi.org/10.1103/PhysRevLett.80.890.

(7) Dudarev, S.; Botton, G. Electron-Energy-Loss Spectra and the Structural Stability of Nickel Oxide: An LSDA+U Study. *Phys Rev B Condens Matter Mater Phys* **1998**, *57* (3), 1505–1509. https://doi.org/10.1103/PhysRevB.57.1505.

(8) Grimme, S. Semiempirical GGA-Type Density Functional Constructed with a Long-Range Dispersion Correction. *J Comput Chem* **2006**, *27* (15), 1787–1799. https://doi.org/10.1002/JCC.20495.

(9) Grimme, S.; Antony, J.; Ehrlich, S.; Krieg, H. A Consistent and Accurate Ab Initio Parametrization of Density Functional Dispersion Correction (DFT-D) for the 94 Elements H-Pu. *Journal of Chemical Physics* **2010**, *132* (15). https://doi.org/10.1063/1.3382344/926936.

(10) Dion, M.; Rydberg, H.; Schröder, E.; Langreth, D. C.; Lundqvist, B. I. Van Der Waals Density Functional for General Geometries. *Phys Rev Lett* **2004**, *92* (24), 246401. https://doi.org/10.1103/PhysRevLett.92.246401.

(11) Lee, K.; Murray, É. D.; Kong, L.; Lundqvist, B. I.; Langreth, D. C. Higher-Accuracy van Der Waals Density Functional. *Phys Rev B Condens Matter Mater Phys* **2010**, *82* (8), 081101. https://doi.org/10.1103/PhysRevB.82.081101.

(12) Klimeš, J.; Bowler, D. R.; Michaelides, A. Chemical Accuracy for the van Der Waals Density Functional. *Journal of Physics Condensed Matter* **2010**, *22* (2), 022201. https://doi.org/10.1088/0953-8984/22/2/022201.

(13) Sun, J.; Ruzsinszky, A.; Perdew, J. Strongly Constrained and Appropriately Normed Semilocal Density Functional. *Phys Rev Lett* **2015**, *115* (3), 036402. https://doi.org/10.1103/PHYSREVLETT.115.036402/FIGURES/1/MEDIUM.

(14) Sun, J.; Remsing, R. C.; Zhang, Y.; Sun, Z.; Ruzsinszky, A.; Peng, H.; Yang, Z.; Paul, A.; Waghmare, U.; Wu, X.; Klein, M. L.; Perdew, J. P. Accurate First-Principles Structures and Energies of Diversely Bonded Systems from an Efficient Density Functional. *Nature Chemistry 2016 8:9* **2016**, *8* (9), 831–836. https://doi.org/10.1038/nchem.2535.

(15) Peng, H.; Yang, Z. H.; Perdew, J. P.; Sun, J. Versatile van Der Waals Density Functional Based on a Meta-Generalized Gradient Approximation. *Phys Rev X* **2016**, *6* (4), 041005. https://doi.org/10.1103/PHYSREVX.6.041005/FIGURES/5/MEDIUM.

(16) Ning, J.; Kothakonda, M.; Furness, J. W.; Kaplan, A. D.; Ehlert, S.; Brandenburg, J. G.; Perdew, J. P.; Sun, J. Workhorse Minimally Empirical Dispersion-Corrected Density Functional with Tests for Weakly Bound Systems: R2SCAN+rVV10. *Phys Rev B* **2022**, *106* (7), 075422. https://doi.org/10.1103/PHYSREVB.106.075422/FIGURES/9/MEDIUM.

(17) Sai Gautam, G.; Carter, E. A. Evaluating Transition Metal Oxides within DFT-SCAN and SCAN+U Frameworks for Solar Thermochemical Applications. *Phys Rev Mater* **2018**, *2* (9), 095401. https://doi.org/10.1103/PHYSREVMATERIALS.2.095401/FIGURES/6/MEDIUM.

(18) Perdew, J. P.; Ruzsinszky, A.; Csonka, G. I.; Vydrov, O. A.; Scuseria, G. E.; Constantin, L. A.; Zhou, X.; Burke, K. Restoring the Density-Gradient Expansion for Exchange in Solids and Surfaces. *Phys Rev Lett* **2008**, *100* (13), 136406. https://doi.org/10.1103/PHYSREVLETT.100.136406/FIGURES/2/MEDIUM.

(19) Krukau, A. V.; Vydrov, O. A.; Izmaylov, A. F.; Scuseria, G. E. Influence of the Exchange Screening Parameter on the Performance of Screened Hybrid Functionals. *Journal of Chemical Physics* **2006**, *125* (22), 35. https://doi.org/10.1063/1.2404663/953719.

(20) Bedzyk, M. Scattering: X-Ray Standing Wave Techniques.

(21) Woodruff, D. P. Surface Structure Determination Using X-Ray Standing Waves. *Reports on Progress in Physics* **2005**, *68* (4), 743–798. https://doi.org/10.1088/0034-4885/68/4/R01.

(22) Duncan, D. A.; Allegretti, F.; Woodruff, D. P. Water Does Partially Dissociate on the Perfect TiO 2(110) Surface: A Quantitative Structure Determination. *Phys Rev B Condens Matter Mater Phys* **2012**, *86* (4). https://doi.org/10.1103/PhysRevB.86.045411.
